# Influence of asthma definition on the asthma-obesity relationship

**DOI:** 10.1186/1471-2458-12-844

**Published:** 2012-10-05

**Authors:** Andrea Antunes Cetlin, Manoel Romeu Gutierrez, Heloísa Bettiol, Marco Antônio Barbieri, Elcio Oliveira Vianna

**Affiliations:** 1 Department of Medicine, Medical School of Ribeirão Preto, University of São Paulo, Ribeirão Preto, São Paulo, Brazil; 2 Department of Pediatrics, Medical School of Ribeirão Preto, University of São Paulo, Ribeirão Preto, São Paulo, Brazil

## Abstract

**Background:**

Epidemiological studies suggest an association between obesity and asthma in adults and children. Asthma diagnosis criteria are different among studies. The aim of this study was to test the influence of asthma definition on the asthma-obesity relationship.

**Methods:**

In a cross-sectional analysis of 1922 men and women, subjects completed a translated questionnaire from the European Community Respiratory Health Survey and underwent spirometry and a bronchial challenge test. Weight, height and waist circumference were measured. Multiple logistic regression analysis was carried out to assess the association of variables related to obesity and asthma. Asthma was defined either by the presence of symptoms with bronchial hyperresponsiveness (BHR) or by a self-report of a physician-made diagnosis. The following variables were separately tested for associations with asthma: socioeconomic characteristics, schooling, physical activity, smoking status, anthropometry and spirometry.

**Results:**

No association was detected between asthma confirmed by BHR and obesity indicators, odds ratio (OR) = 1.08 (95% confidence interval: 0.69 - 1.68) for obesity assessed by body mass index ≥ 30 kg/m^2^; OR = 1.02 (0.74 - 1.40) for obesity assessed by abnormal waist-to-height ratio; and, OR = 0.96 (0.69 - 1.33) for abnormal waist circumference. On the contrary, a previous diagnosis of asthma was associated with obesity, OR = 1.48 (1.01 - 2.16) for body mass index ≥ 30 kg/m^2^; OR = 1.48 (1.13 - 1.93) for abnormal waist-to-height ratio; and, OR = 1.32 (1.00 – 1.75) for abnormal waist circumference. Female gender, schooling ≥ 12 years and smoking were associated with BHR-confirmed asthma. Physically inactive subjects were associated with a previous diagnosis of asthma.

**Conclusions:**

Our findings indicate that the relationship between asthma and obesity in epidemiological studies depends on the definition adopted. Certain components of asthma, for instance, symptoms may be more prone to the obesity influence than other ones, like bronchial hyperresponsiveness.

## Background

Overweight and obesity are major causes of morbidity and mortality. The prevalence of asthma has experienced an increase, with rates that are greater today than they were 30 years ago [1,2]. Different causes for this increase have been postulated. Epidemiological studies have suggested an association between obesity and asthma in adults and children [3-6].

Several cross-sectional studies have found an increase in the prevalence of asthma among obese patients. These studies included a large number of patients and have provided significant epidemiological data on both disorders. However, in most studies, asthma was diagnosed and classified solely on the basis of a compatible clinical history, acceptance of a previous diagnosis, or based on the use of anti-asthma drugs [7-11]. Few studies on the asthma-obesity association employed bronchial challenge tests. Results of these studies are not consistent [12-15].

The bronchial challenge test with methacholine to measure bronchial hyperresponsiveness is considered one of the best approaches to identify asthma in clinical practice. In the last decade, the assessment of bronchial hyperresponsiveness as a tool for asthma diagnosis has become common practice in epidemiological studies, for example, European Community Respiratory Health Survey (ECRHS), the International Study on Allergy and Asthma in Childhood (ISAAC) and Study on Occupational Allergy Risks (SOLAR II) [16]. Approved standardizations of bronchial challenge tests for methacholine have been published. The worldwide use of these protocols allows international comparison of results [17].

In a previous study, we showed that asthma prevalence and asthma risk factors may depend on the asthma definition. Prevalence varied from 3.5% when asthma was defined by asthma attacks in the last 12 months to 19.4% when asthma was defined by wheezing. In that study, maternal asthma and perceived food allergy were related to asthma defined by a score based on 8 questions, but they were not related to the other asthma definitions. Atopy, smoking and rhinitis were risk factors for asthma regardless of the asthma definition [18]. The influence of different asthma definitions on the asthma and obesity relationship has not been addressed.

We hypothesized that the relationship between asthma and obesity may be under the influence of the fact that obesity leads to a set of clinical and physiological perturbations that mimics asthma. In this case, the use of questionnaires to define asthma could induce an association between asthma and obesity due to symptoms that are not asthma-dependent. Therefore, the criteria for asthma detection could have impact on the putative association. We aimed to test the influence of asthma definition on the asthma-obesity relationship by evaluating two asthma definitions, one based on methacholine challenge test and the other one based on a previous physician-made diagnosis of asthma.

## Methods

This is a cross-sectional analysis of a Brazilian population sample. We interviewed 2063 men and women aged 23 to 25 years randomly selected from a cohort of 6827 singleton babies born during the years of 1978 and 1979 in the city of Ribeirão Preto, a regional center in the Northeastern region of São Paulo State, Southeastern Brazil. The main economic activities are sugar cane industry, trading, services, and financing. The number of inhabitants in the city of Ribeirão Preto was approximately 550.000 when data were collected between 2002 and 2004.

### Individuals

The potential participants in the evaluation were identified on the basis of the charts of the liveborns of the original cohort. For the location of the participants the following sources were used: the system of electronic scheduling of visits for users of the public medical services, lists of users of private health plans, charts of evaluation of schoolchildren of the cohort performed in 1987 to 1989, and charts of evaluation of the military recruits belonging to the original cohort. A total of 5665 individuals were located, with the initial contact being made with those who had a fixed or mobile phone, in view of the high distribution of telephones per inhabitant. In addition, letters containing explanations about the objectives of the study and a telephone number reserved for the project were mailed. Other means of dissemination of the information were used, such as television, radio and newspapers, as well as the distribution of explanatory posters to public health services. Based on the geo-economic characterization of the city which is composed of four geographic regions defined by the income of the head of the family and classified as “poor”, “middle-poor”, “middle-rich” and ”rich”, one of the each three individuals belonging to the same geographic area was contacted. In the case of refusal or of the impossibility to participate (e.g., imprisonment, death or serious disease) or the inability to locate an individual, contact was made with the next name on the list.

In this process, 705 individuals had to be excluded because of refusal (209 cases), imprisonment (31 cases), death after 20 years of age (34 cases), and failure to appear for the interview (431 cases). Thus, 2063 adults effectively participated in the study, corresponding to 31.8% of the original sample. We considered original sample as 6827 singleton babies minus 343 deaths before 20 years of age. Of these 2063 subjects, 1922 underwent the bronchial challenge test. We compared those 2063 subjects with our sample of 1922 subjects regarding gender, body mass index (BMI), waist circumference, waist-to-height ratio, physical activity, smoking, schooling and BHR. We did not find significant differences.

### Questionnaire

The ECRHS [19] questionnaire translated into Portuguese and adapted to the Brazilian lexicon was employed to evaluate respiratory symptoms and a previous physician diagnosis of asthma. Four questions of the ECRHS questionnaire were selected: a question about wheezing, about chest tightness, about night-time and daytime breathlessness at rest. The presence of any of these symptoms within the last 12 month in association with bronchial hyperresponsiveness (BHR) was defined as BHR-confirmed asthma. Thus, symptoms reported by non-hyperresponsive individuals were not sufficient to classify them as asthmatic. Another question was asked to determine a previous physician made diagnosis of asthma and a positive answer to this question defined a self-report of a previous physician diagnosis of asthma, irrespective of symptoms or BHR.

We also asked about physical activity status, smoking status and educational level of the subjects. Physical activity status was defined as active if the subject regularly practiced physical activity, including activity at work and inactive when the reply was negative. Regarding smoking status, the subject was asked if he/she had been smoking at least one month before the interview. Those who responded positively were defined as smokers and those who responded negatively as non-smokers. Finally, socioeconomic level was evaluated on the basis of educational level, determined by asking about the numbers of years of schooling and divided into three groups: 8 years or less, 9 to 11 years or 12 years or more. Gender was obtained from the medical interview chart.

### Anthropometric evaluation

Height was measured to the last completed millimeter using a flexible and inextensible tape with the subject standing and barefoot. Supervised university nurses and physicians especially trained for this study undertook all the assessments, including the administered questionnaires. For the analysis of BMI (body mass index = weight/height^2^), the subjects were divided into three groups: normal (BMI ≤ 24.9 kg/m^2^), overweight (BMI between 25.0 and 29.9 kg/m^2^) and obese (BMI ≥ 30 kg/m^2^) [20]. We measured waist circumference and waist-to-height ratio as indicators of central obesity. Waist was measured at the halfway point between the lower margin of the last rib and the iliac crest with an inextensible metric tape. Waist circumference was abnormal when ≥ 94 centimeters for men and ≥ 80 centimeters for women and normal when below these values [21]. The waist-to-height ratio was calculated and defined as abnormal when ≥ 0.5 for both genders and normal when below this value [22].

### Bronchial responsiveness

The bronchial responsiveness to methacholine was measured using the 2 minute tidal breathing method. Increasing concentrations of methacholine (0.06, 0.125, 0.25, 0.5, 1, 2, 4, 8, and 16 mg/mL) were aerosolized with a DeVilbiss 646 nebulizer (Sunrise Medical HHG Inc, Somerset, PA, USA) driven by a computer-activated dosimeter (Koko Digidoser System, PDS Instrumentation, Inc., Louisville, CO, USA) with an output of 9 mL per 0.6 second (total delivery of 0.045 mL). Forced expiratory volume in the first second (FEV_1_) was measured at baseline and 2 minutes after each tidal breathing period. The test was stopped when either a 20% fall in FEV_1_ was achieved or the final concentration was reached. The provocative concentration causing a 20% fall in FEV_1_ (PC_20_) was calculated with Koko software. We considered PC_20_ ≤ 4 mg/mL to indicate BHR. The contraindications of the methacholine challenge test were all conditions that might compromise the quality of the test or that might subject the patient to increased risk or discomfort, including FEV_1_ < 60% of the predicted value, pregnancy, nursing mothers and inability to perform acceptable quality spirometry. During preparation, subjects were questioned about factors that could increase or decrease bronchial responsiveness, such as current respiratory infection or asthma medication. When a factor was present, the tests were postponed [17]. The tests and measurements were carried out in a healthcare setting with easy access to medical facilities.

The study was approved by the institutional ethics committee and all participants signed a consent form after reading and listening to the aims and procedures of the study.

### Statistical analysis

Comparisons between genders were performed using x^2^ statistics for asthma, previous diagnosis of asthma, nutritional status (BMI, waist circumference and waist-to-height ratio), daily physical activities, smoking status and socioeconomic level. Multiple logistic regression analysis was performed to assess the association between nutritional status and asthma confirmed by BHR and asthma defined by self-report of a previous diagnosis. Data were adjusted for confounding variables such as gender, socioeconomic level, smoking status and physical activities. No significant interactions between variables were found. Statistical analyses were performed with StataSE 9.1 [StataCorp, College Station, Texas 2005]. The statistical significance level was set at 0.05.

## Results

Of the 1922 volunteers who completed all tests, 427 (22.2%) had BHR. The prevalence of asthma confirmed by BHR and symptoms was 10.4% and the prevalence of a previous diagnosis of asthma was 14.8%. The prevalence of obesity ranged from 12.1% considering BMI to 34.0% considering waist-to-height ratio (Table 1). The prevalences of asthma-like symptoms of all participants were: 19.1% for wheezing, 12.0% for chest tightness, 11.9% for daytime breathlessness at rest, and 8.8% for nighttime breathlessness. The prevalences of asthma-like symptoms were significantly higher among women than men. For wheezing, the prevalence was 16.9% in men and 21.1% in women (p = 0.018). For chest tightness, breathlessness at rest, night-time breathlessness, the prevalences in men and women were 6.9% and 17.0% (p < 0.001), 6.5% and 17.1% (p < 0.001), 5.2% and 12.2% (p < 0.001), respectively.

**Table 1:** Prevalence of the studied variables according to gender

**Variables**	**Total (N = 1922)**	**Male (N = 942)**	**Female (N = 980)**	**p**
	**n (%)**	**n (%)**	**n (%)**	
Asthma - confirmed by BHR*				< 0.001
No	1722 (89.6)	875 (92.9)	847 (86.4)	
Yes	200 (10.4)	67 (7.1)	133 (13.6)	
Asthma - previous diagnosis**				0.563
No	1638 (85.2)	798 (84.7)	840 (85.7)	
Yes	284 (14.8)	144 (15.3)	140 (14.3)	
**BMI (kg/m**^**2**^**)**^**#**^				< 0.001
< 25.0	1224 (63.8)	532 (56.5)	692 (70.8)	
25.0 - 29.9	464 (24.2)	287 (30.5)	177 (18.1)	
≥ 30.0	231 (12.0)	122 (13.0)	109 (11.1)	
**WC**				< 0.001
Normal	1300 (67.7)	600 (63.7)	700 (71.5)	
Abnormal	621 (32.3)	342 (36.3)	279 (28.5)	
**WHR**^**§**^				< 0.001
Normal (< 0.50)	1268 (66.0)	547 (58.0)	721 (73.7)	
Abnormal (≥ 0.50)	652 (34.0)	395 (42.0)	257 (26.3)	
**Physical Activity**				< 0.001
Active	1113 (57.9)	669 (71.0)	444 (45.3)	
Inactive	809 (42.1)	273 (29.0)	536 (54.7)	
**Smoking habit**				< 0.001
Non-smokers	1588 (82.6)	747 (79.3)	841 (85.8)	
Smokers	334 (17.4)	195 (20.7)	139 (14.2)	
**Schooling (years)**				0.060
< 8	282 (14.7)	146 (15.5)	136 (13.9)	
8-11	978 (50.9)	496 (52.6)	482 (49.1)	
≥ 12	662 (34.4)	300 (31.9)	362 (37.0)	

n: number in that category.

BHR: bronchial hyperresponsiveness.

BMI: body mass index.

WC: waist circumference.

WHR: waist-to-height ratio.

* asthma defined by methacholine PC_20_ ≤ 4 mg/mL (BHR) and symptoms.

** asthma defined by self-report of a previous physician diagnosis.

# two female subjects and one male subject did not have information about BMI.

^§^ two female subjects did not have information about the waist-to-height ratio.

No association has been detected between BHR-confirmed asthma and obesity (Tables 2, 3 and 4 for BMI, waist-to-height ratio and waist circumference, respectively); on the other hand, a previous diagnosis of asthma was associated with obesity (Table 2 for BMI, Table 3 for waist-to-height ratio, and Table 4 for waist circunference). Some variables were associated with asthma confirmed by BHR: female gender, schooling ≥ 12 years and smoking (Tables 2, 3 and 4), but were not associated with asthma diagnosed by the physician. Physically inactive subjects were associated with a previous diagnosis of asthma (Tables 2, 3 and 4).

**Table 2:** Association between asthma and BMI

**Variables**	**Asthma confirmed by BHR**			**Previous asthma diagnosis**		
	**N**	**cases**	**OR (95% CI)**	**N**	**cases**	**OR (95% CI)**
**BMI** (kg/m^2^)	1919	200		1919	284	
< 25.0	1224	128	1	1224	182	1
25.0-29.9	464	44	0.94 (0.65 – 1.37)	464	80	1.38 (1.03 – 1.86)
≥ 30.0	231	28	1.08 (0.69 – 1.68)	231	42	1.48 (1.01 – 2.16)
**Physical activity**	1922	200		1922	284	
Active	1113	97	1	1113	149	1
Inactive	809	103	1.16 (0.85- 1.58)	801	135	1.37 (1.05 – 1.79)
**Gender**	1922	200		1922	284	
Male	942	67	1	942	144	1
Female	980	133	2.15 (1.55 – 2.98)	980	140	0.89 (0.68 – 1.16)
**Smoking habit**	1922	200		1922	284	
Non-smokers	1588	156	1	1588	234	1
Smokers	334	44	1.48 (1.02 – 2.13)	334	50	1.00 (0.71 - 1.40)
**Schooling (years)**	1922	200		1922	294	
≥ 12	282	45	1	282	35	1
9-11	978	118	0.74 (0.50 – 1.08)	978	144	1.27 (0.85 – 1.89)
≤ 8	662	37	0.31 (0.19 – 0.50)	662	105	1.47 (0.97 – 2.23)

N: number of subjects.

cases: numbers of subjects with asthma in every category.

CI: confidence interval.

OR: odds ratio.

BMI: body mass index.

BHR: bronchial hyperresponsiveness.

Analysis was adjusted for gender, socioeconomic level, smoking status and physical activities.

Multivariate analysis.

**Table 3:** Association between asthma and WHR

**Variables**	**Asthma confirmed by BHR**			**Previous asthma diagnosis**		
	**N**	**cases**	**OR (95% CI)**	**N**	**cases**	**OR (95% CI)**
**WHR**	1920	200		1920	284	
< 0.50	1268	131	1	1268	166	1
≥ 0.50	652	69	1.02 (0.74 – 1.40)	652	118	1.48 (1.13 – 1.93)
**Physical activity**	1922	200		1922	284	
Active	1113	97	1	1113	149	1
Inactive	809	103	1.16 (0.85 - 1.58)	801	135	1.35 (1.04 – 1.76)
**Gender**	1922	200		1922	284	
Male	942	67	1	942	144	1
Female	980	133	2.16 (1.56 – 3.01)	980	140	0.90 (0.69 – 1.18)
**Smoking habit**	1922	200		1922	284	
Non-smokers	1588	156	1	1588	234	1
Smokers	334	44	1.47 (1.02 – 2.13)	334	50	1.00 (0.71 - 1.40)
**Schooling (years)**	1922	200		1922	294	
≥ 12	282	45	1	282	35	1
9-11	978	118	0.74 (0.51 – 1.08)	978	144	1.28 (0.86 – 1.91)
≤ 8	662	37	0.36 (0.19 – 0.49)	662	105	1.49 (0.98 – 2.26)

N: number of subjects.

cases: numbers of subjects with asthma in every category.

CI: confidence interval.

OR: odds ratio.

WHR: waist-to-height ratio.

BHR: bronchial hyperresponsiveness.

Analysis was adjusted for gender, socioeconomic level, smoking status and physical activities.

Multivariate analysis.

**Table 4:** Association between asthma and WC

**Variables**	**Asthma confirmed by BHR**			**Previous asthma diagnosis**		
	**N**	**cases**	**OR (95% CI)**	**N**	**cases**	**OR (95% CI)**
**WC**	1921	200		1921	284	
normal	1300	141	1	1300	193	1
abnormal	621	59	0.96 (0.69 – 1.33)	621	91	1.32 (1.00 – 1.75)
**Physical activity**	1922	200		1922	284	
Active	1113	97	1	1113	149	1
Inactive	809	103	1.16 (0.85 - 1.58)	801	135	1.35 (1.04 – 1.76)
**Gender**	1922	200		1922	284	
Male	942	67	1	942	144	1
Female	980	133	2.16 (1.56 – 2.99)	980	140	0.84 (0.65 – 1.09)
**Smoking habit**	1922	200		1922	284	
Non-smokers	1588	156	1	1588	234	1
Smokers	334	44	1.48 (1.02 – 2.14)	334	50	1.01 (0.72 - 1.41)
**Schooling (years)**	1922	200		1922	294	
≥ 12	282	45	1	282	35	1
9-11	978	118	0.74 (0.51 – 1.09)	978	144	1.27 (0.85 – 1.90)
≤ 8	662	37	0.31 (0.19 – 0.49)	662	105	1.47 (0.97 – 2.23)

N: number of subjects.

cases: numbers of subjects with asthma in every category.

CI: confidence interval.

OR: odds ratio.

WC: waist circumference.

BHR: bronchial hyperresponsiveness.

Analysis was adjusted for gender, socioeconomic level, smoking status and physical activities.

Multivariate analysis.

## Discussion

This cross-sectional study was aimed to investigate the influence of asthma definition on the asthma-obesity association. We adopted two very common diagnostic criteria for asthma: 1) symptoms associated with a positive methacholine challenge test; and 2) self-report of physician-diagnosed asthma. We found that the relationship between asthma and obesity in epidemiological studies depends on the criteria adopted. An association was detected between physician-made diagnosis of asthma and obesity, but not between BHR-confirmed asthma and obesity. These findings did not change for any of the variables used to evaluate obesity. In addition, female gender, schooling and smoking were associated with asthma defined by BHR.

One probable interpretation of this observation is the overdiagnosis of asthma in obese people. Because people who are obese are more likely than nonobese people to report dyspnea [23] or asthma-like symptoms, they may be more likely to be misdiagnosed by their physicians as having asthma. Thirty-seven percent of non-asthmatic obese women reported a higher degree of dyspnea during moderate exercise. This exercise-induced breathlessness seemed to be consequence of an elevated oxygen cost of breathing and may explain the overdiagnosis of asthma in obese patients [24]. Obesity is also associated with mechanical respiratory disorders regarding functional residual capacity and tidal volume that could cause respiratory symptoms [25,26].

Aaron et al. [27] conducted a study to determine the proportion of obese Canadian adults who had an incorrect diagnosis of asthma. They found that one-third of the patients who had received a diagnosis of asthma by a physician had no evidence of asthma when their medication was tapered and when they underwent spirometry and bronchial challenge tests. In a recent study [28], among 304 adults who reported physician-diagnosed asthma recruited directly from the community, 83 (27%) had negative methacholine challenges. Authors suggested that there was an incorrect diagnosis of asthma in many of these subjects. Further studies are necessary to evaluate the impact of respiratory symptoms on the overdiagnosis of asthma among obese people in different settings and countries.

Our results could also be interpreted as a consequence of the time interval between past symptoms and study measurements. Natural history of asthma shows variations of asthma severity in a same individual. Data on this subject indicate that a high proportion (up to 50%) of asthmatics experience a clinically defined remission from childhood to adulthood [29-36]. Some of the volunteers in our study may have received an appropriate diagnosis and went into remission before our evaluation. Although such a remission could present with normalized lung function, several studies have documented persisting bronchial hyperresponsiveness in subjects in remission of asthma [30,37-39]. Since BHR-confirmed asthma was based on both symptom and BHR, currently symptom-free patients with BHR would not be considered to have asthma in our protocol.

If the obesity influence on asthma is really dependent on the asthma definition, the evaluation of asthma components separately will reveal different associations with obesity. In fact, respiratory symptoms are clearly affected by obesity in most published studies [25,26,40], whereas BHR and exercise-induced bronchospasm have not been showed to be under the influence of obesity or weight loss in previous studies [41-44].

Schachter et al. reported an analysis of cross sectional data in 1971 Australian adults. Despite the fact that severe obesity was associated with a higher prevalence of wheeze, obese subjects did not have any increase in bronchial responsiveness to histamine [45]. Aaron et al. analyzed spirometry and BHR of 58 obese asthmatic women before and after an intensive 6-month weight loss program. The results showed that FEV_1_ and FVC (forced vital capacity) increased in obese women who lost a significant amount of weight and did not improve in those who failed to lose weight. Findings did not demonstrate any significant effect of weight loss on bronchial responsiveness. The authors suggested that improvements in FEV_1_ and FVC occur due to a reduction in massloading on the respiratory system, rather than improvements in asthma per se [46]. A recent article reported on an analysis of cross-sectional data from 717 Korean adolescents, Yoo et al. tested the effect of increased BMI on the prevalence of allergic diseases, atopy, BHR, and biomarkers of allergic inflammation (eosinophilic count and serum IgE levels). Overweight (BMI > 85^th^ percentile) was associated with an increased prevalence of wheezing and atopy, but it was not associated with augmented bronchial responsiveness, except among girls [47]. An increase in serum inflammatory markers (including white blood cell counts) has been reported in this and in some case–control studies of obese children [47-49]. Then, authors postulated that “BHR seems to represent a different pathway in the association between obesity and asthma” [47]. Several of other studies tested the association between obesity and BHR in patients with asthma; they have not found significant association [50-57].

Airway inflammation is another defining characteristic of asthma [58]. The effect of obesity on asthma inflammation has also been studied by many authors. Although inflammatory pathways are thought to play a role, obesity is not associated with increased eosinophilic inflammation and there is not enough evidence in humans that systemic inflammation, heightened in obesity, regulates asthmatic airway response or symptoms in the obese. Also, the importance of non-eosinophilic airway inflammation needs better understanding. [13,59,60].

There is little information about the trends of asthma in Latin America, although the prevalence of asthma symptoms has been shown to be high. Obesity has markedly increased in Latin America [61]. A recent study with a protocol similar to the one used in our study looked at the association of asthma and obesity in Chile [51]. In that study the authors found a high prevalence of obesity, especially in women, demonstrating that obesity is also a problem in some of the middle industrialized countries. There was a positive association between BMI and asthma symptoms. However, waist circumference was not associated with asthma symptoms and both BMI and waist circumference were negatively associated with BHR. Similar to our study, BHR did not explain the association between asthma and obesity.

BHR and respiratory symptoms, used herein to define asthma, may be found in other diseases than asthma, like chronic obstructive pulmonary disease (COPD). However, since the subjects in our study were young (ranging from 23 to 25 years old) and smoking prevalence was low in this cohort [62], COPD does not seem to be a confounding diagnosis.

The increasing prevalence of asthma is still a matter of preoccupation worldwide [63]. The need for explanation and prophylaxis makes our study relevant to contribute to the understanding of this phenomenon. It is interesting to know the role of obesity as a possible factor increasing asthma prevalence. Defining the component of asthma in which obesity has significant influence will improve the search for mechanisms of the asthma-obesity association. Our observations should lead to future studies to distinctly look for the effects of obesity upon specific characteristics of respiratory diseases, for instance, respiratory symptoms, airway inflammation, lung function and bronchial responsiveness.

## Conclusions

This study added evidence to the asthma-obesity relationship because we tested this association with both definitions: BHR-confirmed asthma and physician-diagnosed asthma. We do not confirm a positive association between obesity and asthma defined by BHR with symptoms. However, the association exists when asthma is defined by a previous diagnosis made by a physician. Further studies are necessary to establish the real diagnosis of asthma that is associated with obesity, as well as to rule out that the association is dependent on the respiratory symptoms caused by obesity.

## Abbreviations

BHR: Bronchial hyperresponsiveness; BMI: Body mass index; COPD: Chronic obstructive pulmonary disease; ECRHS: European Community Respiratory Health Survey; FVC: Forced vital capacity; FEV_1_: Forced expiratory volume in the first second; IgE: Immunoglobulin E; ISAAC: the International Study on Allergy and Asthma in Childhood; OR: Odds ratio; PC_20_: the provocative concentration causing a 20% fall in FEV_1_; SOLAR II: Study on Occupational Allergy Risks.

## Competing interests

The authors declare no competing interests.

## Authors’ contributions

AAC: performed data organization, analysis and interpretation of data, and drafted the manuscript. MRG: performed the statistical analysis, the interpretation and organization of data. HB: participated in the conceiving and designing of the study and data collection MAB: participated in the conceiving and designing of the study and data collection. EOV: participated in the conceiving, planning and structuring of the pulmonary function laboratory and skin tests routine (prick test), interpretation of data and drafted the manuscript. All authors read and approved the final manuscript.

## Pre-publication history

The pre-publication history for this paper can be accessed here:

http://www.biomedcentral.com/1471-2458/12/844/prepub
